# The Relationships Between Lipid Accumulation Product Levels and Cognitive Decline Over 4 Years in a Rural Area of Xi’an, China

**DOI:** 10.3389/fnagi.2021.761886

**Published:** 2021-11-19

**Authors:** Yanyu Wang, Shan Wei, Rong Zhou, Suhang Shang, Liangjun Dang, Ling Gao, Chen Chen, Kang Huo, Jingyi Wang, Jin Wang, Qiumin Qu

**Affiliations:** ^1^Department of Neurology, The First Affiliated Hospital of Xi’an Jiaotong University, Xi’an, China; ^2^Huyi Hospital of Traditional Chinese Medicine, Xi’an, China; ^3^Center for Brain Science, The First Affiliated Hospital of Xi’an Jiaotong University, Xi’an, China

**Keywords:** lipid accumulation product, cognitive decline, risk factor, epidemiology, follow-up

## Abstract

**Background and Aims:** The relationships between blood lipid levels and obesity and cognitive impairment have not been fully determined. Considering that the lipid accumulation product (LAP) is a composite index of blood lipid levels and obesity, we investigated the relationships between LAP levels at baseline and cognitive decline over 4 years.

**Methods:** A total of 983 subjects (≥40 years) from a longitudinal cohort in a village of Xi’an, China, who completed the baseline survey were followed-up for 4 years. All participants underwent face-to-face interviews and cognitive assessments at baseline and at the 4-year follow-up. The Mini-Mental State Examination (MMSE) was used to assess cognitive function, and an MMSE score dropping ≥ 2 points from baseline was defined as cognitive decline. The relationships between LAP and cognitive decline were analyzed by linear regression models.

**Results:** During the 4-year follow-up, 172 patients exhibited cognitive decline (17.5%). Univariate analysis showed that the rate of change in MMSE score was significantly different between the low-LAP group and the high-LAP group (*t* = −2.26, *p* = 0.024). Multiple linear regression indicated that a high LAP was positively associated with cognitive decline (β = 0.564, *p* = 0.012). Stratified multivariate analysis showed that LAP was positively associated with cognitive decline in the normal blood pressure female subgroup (β = 1.29, *p* = 0.002) but not in the high blood pressure group or the male group.

**Conclusions:** High LAP is associated with cognitive decline in females with normal blood pressure but not in those with high blood pressure or males. This indicates that the relationships between blood lipid levels and obesity and cognitive impairment may be affected by blood pressure and sex.

## Introduction

Cognitive impairment has received much attention due to alarming increases in its prevalence, making it a major public health concern exerting societal pressure worldwide. Effective interventions for cognitive impairment are limited (Livingston et al., 2020). Thus, identifying and treating risk factors to prevent cognitive impairment is of great importance.

Obesity has become a social problem and a major contributor to the global burden of disease, as it is associated with an increased risk of numerous chronic diseases, including type 2 diabetes, hypertension, cardiovascular disease, hyperlipidemia, and stroke (Williams et al., 2015). Obesity is also a significant risk factor for dementia (Xu et al., 2011; Alford et al., 2018). According to previous reports, abdominal obesity plays an important role in cognitive decline. Some studies support the detrimental effect of abdominal adiposity measured by waist circumference (WC) or waist-hip ratio (WHR) on cognitive decline (Gustafson et al., 2009; Cui et al., 2013; Gardener et al., 2020). A few surveys reported that visceral adiposity measured by medical imaging methods was associated with poor cognitive function (Isaac et al., 2011; Yoon et al., 2012). Recently, a study from Singapore used the bioelectrical impedance method and agreed with the results (Moh et al., 2020). However, a Brazilian autopsy study showed that abdominal visceral fat was inversely associated with cognitive impairment (Nishizawa et al., 2019). In previous studies by our team, we found that triglycerides (TGs) negatively correlated with cognitive impairment in middle-aged male participants (Zhao et al., 2019), and abdominal obesity measured by WC or WHR alone does not appear to be associated with a higher risk of cognitive impairment (Li et al., 2018).

The lipid accumulation product (LAP) is based on a combination of WC and fasting TG (Kahn, 2005). LAP reflects the combined anatomic and physiologic changes associated with lipid overaccumulation in adults and has been used as a marker of visceral obesity (Kahn, 2005; Kwon and Han, 2019). LAP is a better indicator than body mass index (BMI) for identifying adults at risk for cardiovascular disease (Vieira et al., 2019), which is also associated with an increased risk of metabolic syndrome, diabetes mellitus and hypertension (Wakabayashi, 2014; Mazidi et al., 2018; Nita et al., 2018; Huang et al., 2019). One new study has shown that a high LAP is closely related to mild cognitive impairment (MCI) in type 2 diabetes patients (Yu et al., 2020).

Sex differences in obesity and cognitive function have been well demonstrated (Li et al., 2017), and previous studies showed that blood pressure and central obesity seem to be important factors in cognitive function (Peters et al., 2020). However, a few studies have analyzed the relationship between central obesity and cognitive impairment depending on gender and blood pressure. In order to explore the influence of gender and blood pressure on the relationships between central obesity and cognitive impairment, more studies are necessary that include grouped subjects based on gender and blood pressure. We hypothesized that high LAP is associated with cognitive decline, particularly in females and in those with hypertension. We studied the relationships between LAP levels and cognitive decline over 4 years. We explored that if high LAP was correlated with cognitive decline and if the correlation was regulated by both sex and blood pressure.

## Materials and Methods

### Participants and Follow-up

This was a population-based longitudinal cohort study. The cohort was conducted from October 2014 through March 2015. We used a stratified, multistage, cluster-sampling methodology to select the study population in rural Xi’an. All residents living in the selected villages were included in the study. The inclusion criteria were as follows: (1) above 40 years old; (2) living in Qubao Village for more than 3 years; (3) without cognitive impairment at baseline; and (4) willing to participate in the study and provide informed consent.

Exclusion criteria were: (1) had a history of stroke; (2) had suffered from diseases that can cause cognitive impairment including infection, nervous system tumor, inflammation of central nervous system (CNS), epilepsy, brain trauma, Parkinson’s disease, and hypothyroidism; (3) had suffered from severe psychopathy, including schizophrenia, bipolar disorder, severe depression or anxiety; (4) had severe cardiac, pulmonary, hematological, liver or kidney dysfunction; (5) did not complete MMSE score, biomarkers, or covariates at baseline.

Four years later, all participants who completed the baseline survey were followed-up from January 2019 to March 2019 using the same method as in the baseline survey.

### Questionnaire Survey

All participants underwent a standardized face-to-face interview for the baseline investigation and follow-up. The interviewers consisted of neurologists and medical students. All interviewers received 1 week of training for the correct use of a unified questionnaire, standardized survey terms, assessment of cognition and community practice, and the consistency between the examiners (kappa: 0.76–1) was evaluated afterward.

Standardized questionnaires were used to collect participant sociodemographic information (sex, age, education, and marital status), lifestyle information (smoking, drinking and physical activity), and medical history (stroke, diabetes, hypertension, coronary heart disease, and dyslipidemia). Additionally, all subjects accepted a general examination, including height, weight, waist circumference, and hip circumference measured in light clothing and with no shoes, by nurses. Blood pressure (BP) was measured on the right arms of patients by nurses using a mercury manometer. BP was measured in the morning before breakfast after a 10-min rest period. The sitting BP was measured twice, and the average of the two measurements was recorded.

The standard of high blood pressure is that SBP is greater than or equal to 140 mmHg and/or DBP is greater than or equal to 90 mmHg. BMI was calculated by dividing weight in kilograms by height in meters squared (kg/m^2^).

### Cognitive Assessment

Global cognitive function was evaluated with the Mini-Mental State Examination (MMSE) in a quiet room by an examiner who accepted uniform training before the study. MMSE scores lower than the cutoff value were used as the criterion for cognitive impairment. The cutoff score of the MMSE was defined as ≤ 17 for illiteracy, ≤ 20 for primary school, and ≤ 24 for junior high school level or above (Katzman et al., 1988).

The change in MMSE score from baseline after 4 years of follow-up was used to measure cognitive decline. Rate of change in MMSE score = (MMSE score at baseline—MMSE score at follow-up)/interval between follow-up visits (years). Dropping points ≥ 2 points was defined as cognitive decline, while dropping points < 2 points was defined as cognitively stable.

### Laboratory Evaluation

From each subject, 6 ml of fasting blood was collected in a purple-top EDTA anticoagulation tube and a red-top non-anticoagulation tube. The blood in the red-top tube was promptly subjected biochemical tests, such as high-density lipoprotein cholesterol (HDL-c), low-density lipoprotein cholesterol (LDL-c), triglycerides (TG), total cholesterol (TC), and fasting blood glucose (FBG). The ApoE genotype was assessed by identifying two polymorphic sites at amino acid residues 112 and 158, which define the ApoE genotype, from DNA extracted from frozen EDTA–anticoagulant blood according to the manufacturer’s protocol (Mahley and Rall, 2000). Subjects were classified as having the ApoEε4 genotype (carrier/non-carrier) according to amyloid status.

The LAP index was calculated according to the following equations: LAP = [WC (cm) − 65] × TG (mmol/L) for males and [WC (cm) − 58] × TG (mmol/L) for females (Kahn, 2005).

### Statistical Analyses

After testing the distribution of each variable, we reported means ± standard deviations for variables with an approximately normal distribution, medians (25% percentile, 75% percentile) for severely skewed continuous variables, and frequencies and percentages for categorical variables. In univariate analyses, independent-sample *t* tests were used for approximately normal distribution, Mann–Whitney *U* tests were used for severely skewed continuous variable distributions, and χ^2^ tests were used to analyze categorical variables. Based on the median value of the LAP distribution, the total study population was divided into two groups. In multivariate analysis, multiple linear regression was used to investigate statistical significance after adjusting for aforementioned covariates. We built two models because of the collinearity between TC and LDL-c. Model 1 used LDL-c and the other confounding factors mentioned above. Model 2 used TC and other confounding factors.

SPSS 26.0 software was used for statistical analysis. A *p* value < 0.05 (two-tailed) was considered to be statistically significant.

## Results

### Characteristics of the Population at Baseline

**FIGURE 1:**
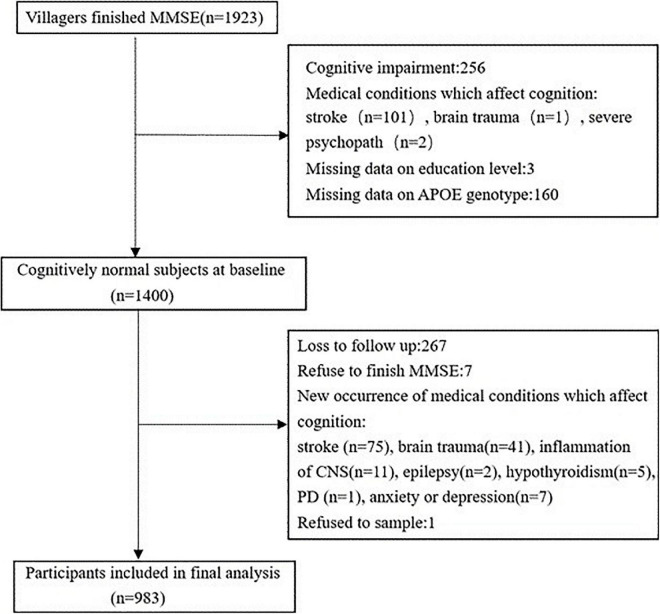
Flow chart of participant selection.

A total of 1,400 subjects were included at baseline. Four years later, 267 individuals (19.07%) were lost to follow-up. Additionally, 150 participants who refused to participate in the survey, and with new occurrence of comorbidities which might contribute to cognitive impairment were further excluded. Finally, 983 participants were included in the analysis (Figure 1).

The characteristics of the total study population at follow-up were shown in Table 1, there were significant differences in age, sex, smoking, hypertension, diabetes mellitus, hyperlipidemia, hip circumstance, WC, BMI, SBP, DBP, FBG, TG, TC, HDL-C, LDL-C, ApoEε4 carrier status and rate of change in MMSE score between the low-LAP group and the high-LAP group.

**TABLE 1:** Characteristics of the participants.

	**Total (*n* = 983)**	**Low LAP (*n* = 492)**	**High LAP (*n* = 491)**	***p***
Age (years)	53.85 ± 9.04	52.84 ± 8.88	54.87 ± 9.1	0.000
Female (n, %)	587 (59.72)	276 (56.10)	311 (63.34)	0.021
Education (years)	8 (4)	8 (3)	7 (5)	0.404
Medical history				
Smoking (n, %)	286 (29.09)	160 (32.52)	126 (25.66)	0.018
Alcohol consumption (n, %)	144 (14.65)	80 (16.26)	64 (13.03)	0.153
Lack of physical activity (n, %)	129 (13.12)	56 (11.38)	73 (14.89)	0.106
Cardiovascular disease (n, %)	39 (4)	16 (3.25)	23 (4.68)	0.250
Hypertension (n, %)	447 (45.47)	161 (32.72)	286 (58.25)	0.000
Diabetes mellitus (n, %)	103 (10.48)	26 (5.28)	77 (15.68)	0.000
Hyperlipidemia (n, %)	473 (48.12)	116 (23.58)	357 (72.71)	0.000
Pulse rate (times/min)	75.25 ± 9.03	75.18 ± 9.83	75.32 ± 8.18	0.810
Waist circumference (cm)	85.13 ± 8.67	80.39 ± 6.88	89.89 ± 7.61	0.000
Hip circumstance (cm)	96.59 ± 6.46	93.78 ± 5.47	99.41 ± 6.16	0.000
BMI (kg/m^2^)	25.37 ± 3.21	23.95 ± 2.64	26.80 ± 3.10	0.000
SBP (mmHg)	130.38 ± 17.36	126.12 ± 15.75	134.64 ± 17.86	0.000
DBP (mmHg)	81.68 ± 9.76	78.88 ± 8.63	84.49 ± 10.03	0.000
Laboratory tests				
FBG (mmol/L)	5.4 (0.73)	5.31 (0.64)	5.49 (0.87)	0.000
TG (mmol/L)	1.41 (0.95)	1.025 (0.43)	1.88 (0.89)	0.000
TC (mmol/L)	4.96 ± 0.92	4.73 ± 0.81	5.18 ± 0.97	0.000
HDL-C (mmol/L)	1.39 ± 0.30	1.49 ± 0.3	1.29 ± 0.28	0.000
LDL-C (mmol/L)	3.25 ± 0.82	3.03 ± 0.73	3.47 ± 0.85	0.000
ApoEε4 (n, %)	137 (13.94)	56 (11.38)	81 (16.50)	0.021
MMSE score	27 (3)	28 (3)	27 (4)	0.079
Cognitive decline (n, %)	172 (17.50)	76 (15.45)	96 (19.55)	0.090
Rate of change in MMSE score (points/year)	−0.09 ± 0.55	0.13 ± 0.53	−0.05 ± 0.58	0.024

*Unpaired Student’s *t*-test and mean ± SD were used to compare the difference of the approximately normally distributed continuous variables between low LAP and high LAP.*

*Mann–Whitney *U* test and median (quartile) were used for the skew distributional data and Chi square and percentage were used for categorical variables.*

*Data are mean (SD), median (interquartile range), or number (percentage).*

*LAP, lipid accumulation product; BMI, body mass index; SBP, systolic blood pressure; DBP, diastolic blood pressure; FBG, fast blood glucose; TC, total cholesterol; TG, triglyceride; HDL-c, high-density lipoprotein; LDL-c, low-density lipoprotein; ApoE, apolipoprotein E; MMSE, Mini-Mental State Examination.*

### Univariate Analysis of Cognitive Decline in the Total Population

During the 4-year follow-up, 172 patients (17.5%) met the criteria of cognitive decline. As shown in Table 2, the cognitive decline group was older, had a lower education level and had more MMSE score decline than the cognitively stable group. There was no significant difference in other variables.

**TABLE 2:** Comparison of Cognitive stable and Cognitive decline group in total study population.

	**Cognitive stable (*n* = 811)**	**Cognitive decline (*n* = 172)**	***p***
Age (years)	53.51 ± 8.88	55.48 ± 9.65	0.009
Female (n, %)	479 (59.06)	108 (62.79)	0.365
Education (years)	8 (4)	7 (5)	0.037
Medical history			
Smoking (n, %)	240 (29.59)	46 (26.74)	0.455
Alcohol consumption (n, %)	119 (14.67)	25 (14.53)	0.963
Lack of physical activity (n, %)	110 (13.56)	19 (11.05)	0.375
Cardiovascular disease (n, %)	30 (3.70)	9 (5.23)	0.349
Hypertension (n, %)	365 (45.01)	82 (47.67)	0.523
Diabetes mellitus (n, %)	84 (10.36)	19 (11.05)	0.789
Hyperlipidemia (n, %)	384 (47.35)	89 (51.74)	0.295
Pulse rate (times/min)	75.25 ± 9.16	75.26 ± 8.42	0.988
Waist circumference (cm)	85.18 ± 8.89	84.9 ± 7.56	0.702
Hip circumstance (cm)	96.64 ± 6.54	159.7 ± 7.27	0.605
BMI (kg/m^2^)	25.37 ± 3.25	25.38 ± 3.01	0.986
LAP	32.96 (31.79)	35.76 (27.46)	0.474
SBP (mmHg)	130.17 ± 17.32	131.35 ± 17.53	0.416
DBP (mmHg)	81.71 ± 9.80	81.56 ± 9.61	0.854
Laboratory tests			
FBG (mmol/L)	5.4 (0.73)	5.4 (0.72)	0.853
TG (mmol/L)	1.4 (0.96)	1.48 (0.85)	0.318
TC (mmol/L)	4.94 ± 0.92	5.02 ± 0.92	0.309
HDL-C (mmol/L)	1.40 ± 0.31	1.38 ± 0.27	0.723
LDL-C (mmol/L)	3.24 ± 0.82	3.31 ± 0.81	0.282
APOE ε (n, %)	117 (14.43)	20 (11.63)	0.336
Rate of change in MMSE score (points/year)	−0.26 ± 0.40	0.74 ± 0.41	0.000

*Unpaired Student’s *t*-test and mean ± SD were used to compare the difference of the approximately normally distributed continuous variables between the Cognitive stable and Cognitive decline group.*

*Mann–Whitney *U* test and median (quartile) were used for the skew distributional data and Chi square and percentage were used for categorical variables.*

*Data are mean (SD), median (interquartile range), or number (percentage).*

*LAP, lipid accumulation product; BMI, body mass index; SBP, systolic blood pressure; DBP, diastolic blood pressure; FBG, fast blood glucose; TC, total cholesterol; TG, triglyceride; HDL-c, high-density lipoprotein; LDL-c, low-density lipoprotein; ApoE, apolipoprotein E; MMSE, Mini-Mental State Examination.*

### Association Between Lipid Accumulation Product and Cognitive Decline in the Total Population

The prevalence of cognitive decline was higher in the high-LAP group than in the low-LAP group; however, this difference was not statistically significant (19.6 vs. 15.4%, *p* = 0.09). The MMSE score at baseline was not significantly different between the low-LAP group and the high-LAP group (*p* = 0.079); however, rate of change in MMSE score was more significant in the high-LAP group than in the low-LAP group (*p* = 0.024) (Table 1).

To exclude the influence of confounding factors on the relationships between LAP and cognitive decline, multiple linear regression models were used. After adjustment for confounding factors, higher LAP was positively associated with cognitive decline (Model 1, β = 0.565, *p* = 0.012; Model 2, β = 0.564, *p* = 0.012) (Table 3).

**TABLE 3:** Multiple linear regression of LAP levels and rate of change in MMSE score.

	**Model**	**β**	**S. E**	**t**	***p***
Total population	1	0.140	0.056	2.499	0.013
	2	0.140	0.056	2.495	0.013
Female	3	0.202	0.075	2.683	0.008
	4	0.201	0.075	2.675	0.008
Male	3	0.014	0.084	0.162	0.871
	4	0.014	0.084	0.164	0.870
Normal BP	5	0.211	0.075	2.805	0.005
	6	0.211	0.075	2.811	0.005
High BP	5	0.019	0.087	0.217	0.829
	6	0.018	0.087	0.202	0.840
Normal BP -female	7	0.239	0.074	3.218	0.001
Normal BP -male	7	–0.071	0.100	–0.708	0.480
High BP -female	7	0.069	0.098	0.700	0.484
High BP -male	7	–0.051	0.102	–0.499	0.618

β, *the unstandardized regression coefficient; S.E, standard deviation.*

*Model 1, adjust for age, gender, education, smoking, lack of physical activity, cardiovascular disease, hypertension, diabetes mellitus, hyperlipidemia, waist circumference, hip circumstance, BMI, SBP, DBP, FBG, TG, HDL-C, LDL-C, and ApoE ε4 genotype.*

*Model 2, adjust for age, gender, education, smoking, lack of physical activity, cardiovascular disease, hypertension, diabetes mellitus, hyperlipidemia, waist circumference, hip circumstance, BMI, SBP, DBP, FBG, TG, TC, HDL-C and ApoE ε4 genotype.*

*Model 3, adjust for age, education, smoking, lack of physical activity, cardiovascular disease, hypertension, diabetes mellitus, hyperlipidemia, waist circumference, hip circumstance, BMI, SBP, DBP, FBG, TG, HDL-C, LDL-C, and ApoE ε4 genotype.*

*Model 4, adjust for age, education, smoking, lack of physical activity, cardiovascular disease, hypertension, diabetes mellitus, hyperlipidemia, waist circumference, hip circumstance, BMI, SBP, DBP, FBG, TG, TC, HDL-C and ApoE ε4 genotype.*

*Model 5, adjust for age, gender, education, smoking, lack of physical activity, cardiovascular disease, diabetes mellitus, hyperlipidemia, waist circumference, hip circumstance, BMI, SBP, DBP, FBG, TG, HDL-C, LDL-C, and ApoE ε4 genotype.*

*Model 6, adjust for age, gender, education, smoking, lack of physical activity, cardiovascular disease, diabetes mellitus, hyperlipidemia, waist circumference, hip circumstance, BMI, SBP, DBP, FBG, TG, TC, HDL-C and ApoE ε4 genotype.*

*Model 7, adjust for age, education, smoking, lack of physical activity, diabetes mellitus, hyperlipidemia, BMI, and ApoE ε4 genotype.*

*BMI, body mass index; SBP, systolic blood pressure; DBP, diastolic blood pressure; FBG, fast blood glucose; TC, total cholesterol; TG, triglyceride; HDL-c, high-density lipoprotein; LDL-c, low-density lipoprotein; ApoE, apolipoprotein E.*

### The Effects of Blood Pressure and Sex on Lipid Accumulation Product and Cognitive Decline

Taking into account the significant difference in abdominal obesity by sex, we performed sex-stratified analyses. After stratification by sex, females (*n* = 587) were younger, showed lower levels of education, smoking, alcohol consumption, physical activity and MMSE scores, and they were more likely to have high pulse rates, WC, LAP, TC, and HDL-C (Table 4). Considering that high blood pressure is an important risk factor for cerebral vascular disease and cognitive impairment, we performed stratified analyses by blood pressure. As shown in Table 5, participants with high blood pressure (*n* = 404) were older, had lower levels of education and MMSE scores, were more likely to have diabetes mellitus and hyperlipidemia, and had high levels of hip circumference, WC, BMI, LAP, SBP, DBP, FBG, TG, TC, and LDL-c.

**TABLE 4:** Comparison of Female group and Male group in total study population.

	**Female (*n* = 587)**	**Male (*n* = 396)**	***p***
Age (years)	53.38 ± 8.64	54.56 ± 9.59	0.049
Education (years)	7 (4)	8 (3)	0.000
Medical history (n, %)			
Smoking (n, %)	5 (0.85)	281 (70.96)	0.000
Alcohol consumption (n, %)	10 (1.70)	134 (33.84)	0.000
Lack of physical activity (n, %)	93 (15.84)	36 (9.09)	0.002
Cardiovascular disease (n, %)	23 (3.92)	16 (4.04)	0.923
Hypertension (n, %)	264 (44.97)	183 (46.21)	0.702
Diabetes mellitus (n, %)	66 (11.24)	37 (9.34)	0.340
Hyperlipidemia (n, %)	276 (47.02)	197 (49.75)	0.401
Pulse rate (times/min)	76.06 ± 8.69	74.06 ± 9.41	0.001
Waist circumference (cm)	83.81 ± 8.66	87.09 ± 8.31	0.000
Hip circumstance (cm)	96.35 ± 6.76	96.95 ± 6.00	0.157
BMI (kg/m^2^)	25.47 ± 3.39	25.22 ± 2.92	0.219
LAP	35.15 (32.12)	30.9 (28.89)	0.001
SBP (mmHg)	130.48 ± 18.26	130.23 ± 15.94	0.820
DBP (mmHg)	81.6 ± 9.89	81.8 ± 9.59	0.761
Laboratory tests			
FBG (mmol/L)	5.41 (0.76)	5.395 (0.69)	0.287
TG (mmol/L)	1.41 (0.91)	1.41 (1.40)	0.945
TC (mmol/L)	5.01 ± 0.94	4.88 ± 0.89	0.040
HDL-C (mmol/L)	1.45 ± 0.31	1.30 ± 0.28	0.000
LDL-C (mmol/L)	3.25 ± 0.83	3.25 ± 0.80	0.915
APOE ε (n, %)	81 (13.80)	56 (14.14)	0.879
MMSE score	27 (4)	28 (3)	0.001
Cognitive decline (n, %)	108 (18.40)	64 (16.16)	0.365

*Unpaired Student’s *t*-test and mean ± SD were used to compare the difference of the approximately normally distributed continuous variables between female and male.*

*Mann–Whitney *U* test and median (quartile) were used for the skew distributional data and Chi square and percentage were used for categorical variables.*

*Data are mean (SD), median (interquartile range), or number (percentage).*

*LAP, lipid accumulation product; BMI, body mass index; SBP, systolic blood pressure; DBP, diastolic blood pressure; FBG, fast blood glucose; TC, total cholesterol; TG, triglyceride; HDL-c, high-density lipoprotein; LDL-c, low-density lipoprotein; ApoE, apolipoprotein E; MMSE, Mini-Mental State Examination.*

**TABLE 5:** Comparison of Normal blood pressure group and High blood pressure group in total study population.

	**Normal blood pressure (*n* = 579)**	**High blood pressure (*n* = 404)**	***p***
Age (years)	51.77 ± 8.46	56.84 ± 9.0	0.000
Female (n, %)	353 (60.97)	234 (57.92)	0.338
Education (years)	8 (4)	7 (5)	0.006
Medical history			
Smoking (n, %)	171 (29.53)	115 (28.47)	0.717
Alcohol consumption (n, %)	88 (15.20)	56 (13.86)	0.560
Lack of physical activity (n, %)	71 (12.26)	58 (14.36)	0.339
Cardiovascular disease (n, %)	19 (3.28)	20 (4.95)	0.187
Diabetes mellitus (n, %)	51 (8.81)	52 (12.87)	0.041
Hyperlipidemia (n, %)	249 (43.01)	224 (55.45)	0.000
Pulse rate (times/min)	74.8 ± 8.90	75.9 ± 9.19	0.062
Waist circumference (cm)	83.64 ± 8.09	87.26 ± 9.03	0.000
Hip circumstance (cm)	95.6 ± 5.76	98.01 ± 7.12	0.000
BMI (kg/m^2^)	24.82 ± 2.88	26.17 ± 3.49	0.000
LAP	27.72 (26.49)	42.01 (36.32)	0.000
SBP (mmHg)	119.63 ± 9.45	145.78 ± 14.22	0.000
DBP (mmHg)	76.23 ± 6.02	89.49 ± 8.74	0.000
Laboratory tests			
FBG (mmol/L)	5.33 (0.72)	5.47 (0.76)	0.000
TG (mmol/L)	1.27 (0.8)	1.63 (0.97)	0.000
TC (mmol/L)	4.89 ± 0.92	5.05 ± 0.92	0.009
HDL-C (mmol/L)	1.40 ± 0.30	1.38 ± 0.31	0.258
LDL-C (mmol/L)	3.20 ± 0.81	3.32 ± 0.82	0.025
APOE ε (n, %)	85 (14.68)	52 (12.87)	0.420
MMSE score	28 (3)	27 (4)	0.004
Cognitive decline (n, %)	104 (17.96)	68 (16.83)	0.646

*Unpaired Student’s *t*-test and mean ± SD were used to compare the difference of the approximately normally distributed continuous variables between normal blood pressure group and high blood pressure group.*

*Mann–Whitney *U* test and median (quartile) were used for the skew distributional data and Chi square and percentage were used for categorical variables.*

*Data are mean (SD), median (interquartile range), or number (percentage).*

*LAP, lipid accumulation product; BMI, body mass index; SBP, systolic blood pressure; DBP, diastolic blood pressure; FBG, fast blood glucose; TC, total cholesterol; TG, triglyceride; HDL-c, high-density lipoprotein; LDL-c, low-density lipoprotein; ApoE, apolipoprotein E; MMSE, Mini-Mental State Examination.*

### Association of Lipid Accumulation Product and Cognitive Decline Stratified by Blood Pressure and Sex

To exclude the influence of sex and BP on the relationships between LAP and cognitive decline, a stratified analysis was used. Univariate analysis showed that rate of change in MMSE score was lower in the low-LAP group than in the high-LAP group (*t* = −3.118, *p* = 0.002) in the female group but not in the male group. Additionally, rate of change in MMSE score was significantly different between the low-LAP group and the high-LAP group in the normal blood pressure group (*t* = −2.567, *p* = 0.011) but not in the high blood pressure group.

Stratified multiple linear regression analysis showed that after controlling for possible confounding factors, LAP positively correlated with cognitive decline in the female group but not in the male group (Table 3). Additionally, LAP was positively related to cognitive decline in the normal blood pressure group but not in the high blood pressure group (Table 3). Multivariate analysis stratified by sex and blood pressure showed that LAP was associated with cognitive decline in the normal blood pressure female subgroup but not in any other subgroup (Table 3).

## Discussion

In this community-based longitudinal cohort study, we investigated the relationships between LAP at baseline and cognitive decline over 4 years and found that LAP was positively associated with ΔMMSE scores. However, the association between LAP and cognitive decline was only found in participants who were female and had normal blood pressure and not in those who were male and had high blood pressure. These results indicate that the relationships between LAP and cognitive decline may be confounded by blood pressure and sex.

The relationships between dyslipidemia and cognitive decline have not been clarified. Some studies have reported that dyslipidemia is likely to have important effects on cognitive decline (van den Kommer et al., 2009; Reis et al., 2013), but others have reported conflicting results (Mielke et al., 2005; Benito-Leon et al., 2015). In the same way, the results regarding the relationships between obesity and cognitive impairment are conflicting (Xu et al., 2011; Qizilbash et al., 2015; Alford et al., 2018). However, LAP, as a composite indicator that can reflect the comprehensive situation of blood lipids and obesity, is a relatively stable indicator to reflect lipid overaccumulation rather than blood lipids (Kahn, 2005). In this study, we investigated the relationships between LAP levels and cognitive decline and found that LAP was positively associated with cognitive decline.

However, the relationships between LAP and cognitive decline were only found in normal blood pressure females but not in male and high blood pressure subjects. The reasons for this are not fully understood. Typical sex differences have been found in cognitive performance, and some studies suggest that cognitive decline was faster in females than in males (Li and Singh, 2014; Alzheimer’s, 2015). A possible underlying mechanism is that sex hormone levels drop during the postmenopausal period, which attenuates the beneficial active effects of hormones in the adult brain and then contributes to cognitive decline (Geerlings et al., 2001). Compared to males, central obesity in females is positively associated with inflammation and insulin resistance (Ahonen et al., 2012; Moser and Pike, 2016; Arnold et al., 2018; Maria Teixeira et al., 2020), and all of these factors working together may have contributed to cognitive decline. In addition, sex differences in obesity have been well demonstrated (Li and Singh, 2014).

Hypertension is the most important risk factor for cerebral vascular disease and cognitive impairment. Many studies have found that elevated blood pressure levels at midlife may be related to the development and progression of Alzheimer’s disease later in life (Walker et al., 2017, 2019). According to a recent study, hypertension is associated with an increased Aβ burden in the brain (Langbaum et al., 2012). Our previous studies have found that elevated blood pressure is positively correlated with cognitive impairment in middle-aged individuals (Shang et al., 2016) and associated with increased plasma Aβ_1–40_ levels in middle-aged individuals and elderly individuals (She et al., 2021). Therefore, we suspect that high blood pressure may overpower the relationships between LAP and cognitive decline. Thus, the relationships between LAP and cognitive decline were found only in normal blood pressure subjects and not in high blood pressure subjects.

The mechanisms of cognitive decline due to abdominal obesity are unclear, and the possible mechanisms are explored here. Excess visceral adipose tissue affects lipid metabolism and the development of chronic inflammation (Blachnio-Zabielska et al., 2018). Adipocytes secrete cytokines in adipose tissue (Galic et al., 2010), such as adiponectin and leptin, which increase insulin sensitivity and change cognitive function directly or indirectly (Kaser et al., 2008; Forny-Germano et al., 2018). The increased macrophages secrete numerous cytokines and chemokines, such as TNF-α, IL-1, and IL-6 (Xu et al., 2003; Glass and Olefsky, 2012), and these cytokines lead to insulin resistance. Moreover, inflammation influences neuronal health *via* its interactions with oxidative stress (Miller and Spencer, 2014; Radi et al., 2014). These effects contribute to mitochondrial dysfunction (Prakash and Kumar, 2014), neurodegeneration (Uehara et al., 2006), the accumulation of β-amyloid in the brain (Farris et al., 2004), and brain atrophy (Mullins et al., 2017). All of these factors lead to a decline in cognitive performance.

Some limitations of this study should be noted. First, LAP was measured only once at baseline. It did not represent the dynamic changes over 4 years. Long-term follow-up and dynamic monitoring of LAP are necessary. Second, the MMSE was used to assess cognitive changes, and a decline ≥ 2 points from baseline was defined as cognitive decline. However, this does not mean that the subjects met the criteria for dementia or MCI. Additionally, the cognitive declines need to be followed-up. Third, the Montreal Cognitive Assessment (MoCA) has higher sensitivity and ceiling than MMSE, that helps better detecting the early stages of cognitive impairment (Roalf et al., 2017), thus, in the further investigation, we would like to assess cognitive function measured by MMSE and MoCA. What’s more, due to the relatively small sample size, it was difficult to investigate whether the effects of LAP on cognitive decline were dose dependent.

## Conclusion

In this population-based longitudinal study, we demonstrated that high LAP is associated with cognitive decline, which was only found in normal blood pressure females but not in those with hypertension or males. This indicates that the relationships between blood lipid levels and obesity and cognitive impairment may be affected by hypertension and sex.

## Data Availability Statement

The raw data supporting the conclusions of this article will be made available by the authors, without undue reservation.

## Ethics Statement

The studies involving human participants were reviewed and approved by Medical Ethics Committee of the First Affiliated Hospital of Xi’an Jiaotong University. The patients/participants provided their written informed consent to participate in this study.

## Author Contributions

YW took part in the survey, did the statistical analysis and wrote the manuscript. SW, RZ, LD, and LG participated in the questionnaire survey and sample collection. SS designed this study and participated in the questionnaire survey and sample collection. CC, KH, JW, and JYW took part in the survey and collected samples. QQ provided technical guidance in all stages of the study. All authors have read and approved the final manuscript.

## Conflict of Interest

The authors declare that the research was conducted in the absence of any commercial or financial relationships that could be construed as a potential conflict of interest.

## Publisher’s Note

All claims expressed in this article are solely those of the authors and do not necessarily represent those of their affiliated organizations, or those of the publisher, the editors and the reviewers. Any product that may be evaluated in this article, or claim that may be made by its manufacturer, is not guaranteed or endorsed by the publisher.
