# Supplementary material for: The Relationships Between Lipid Accumulation Product Levels and Cognitive Decline Over 4 Years in a Rural Area of Xi’an, China
Source: Front Aging Neurosci. 2021 Nov 19;13:761886. doi: 10.3389/fnagi.2021.761886 (PMC8640205; doi:10.3389/fnagi.2021.761886)
Supplement: Supplementary file 1 [file Data_Sheet_1.docx]

**Appendix**

(residual plots)

1.Total
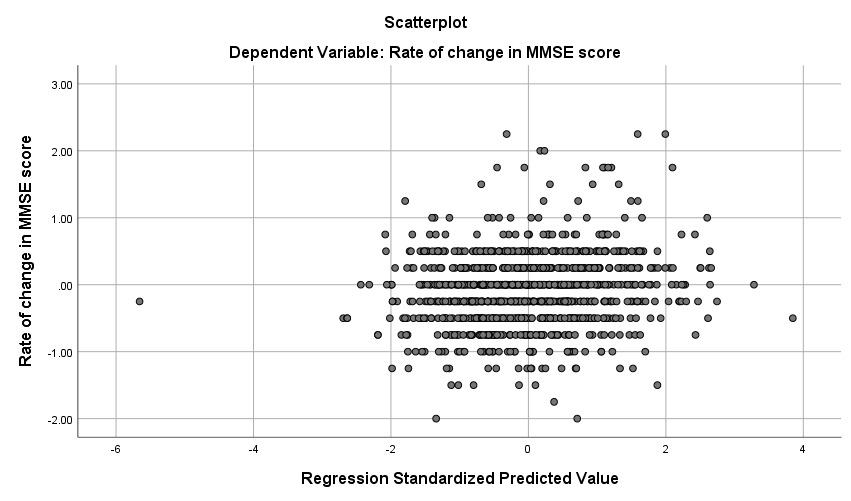

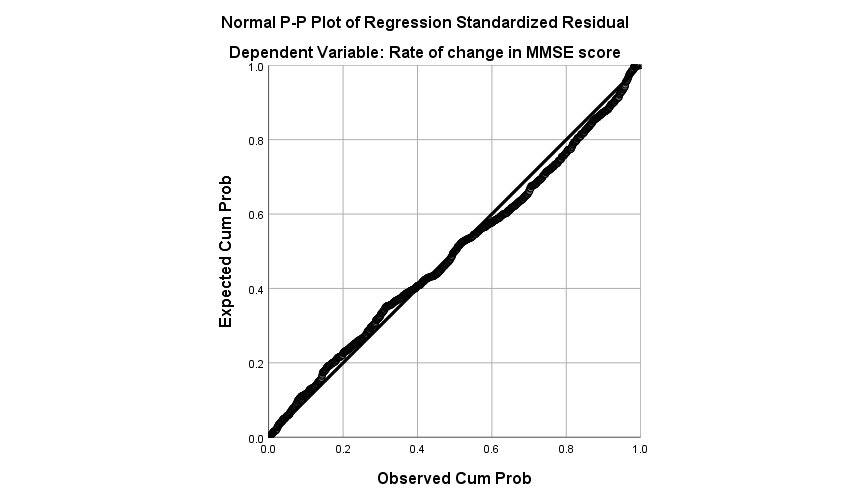


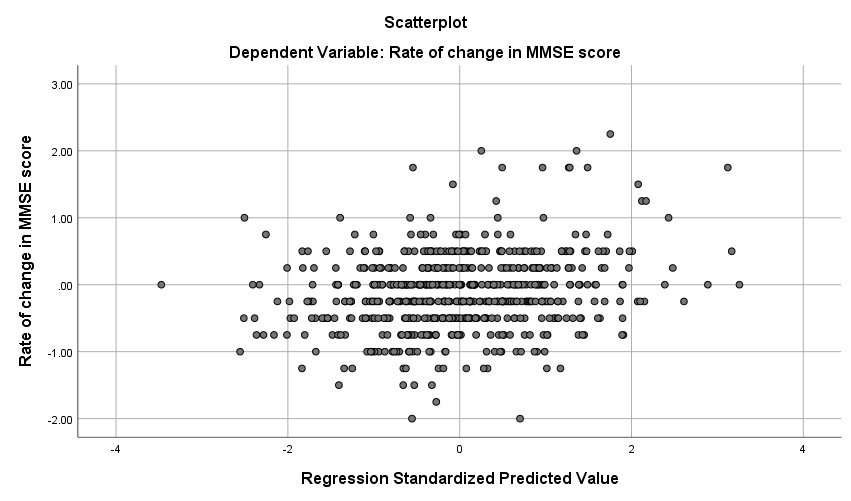

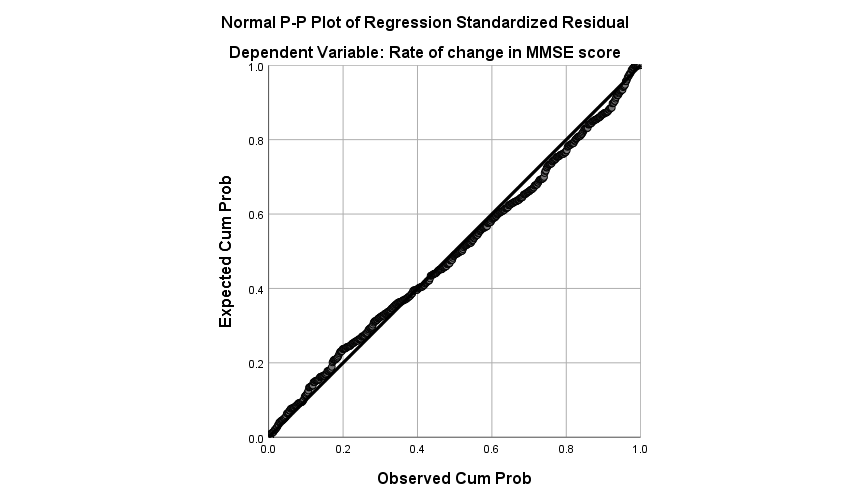
2.Female


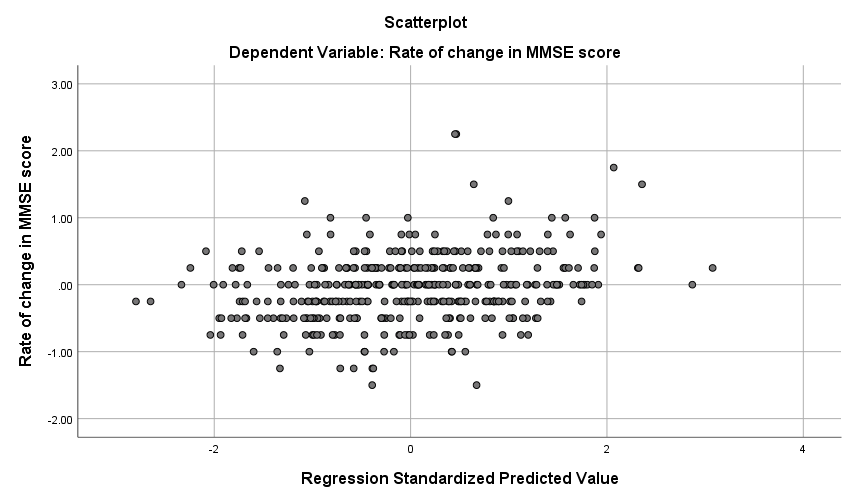

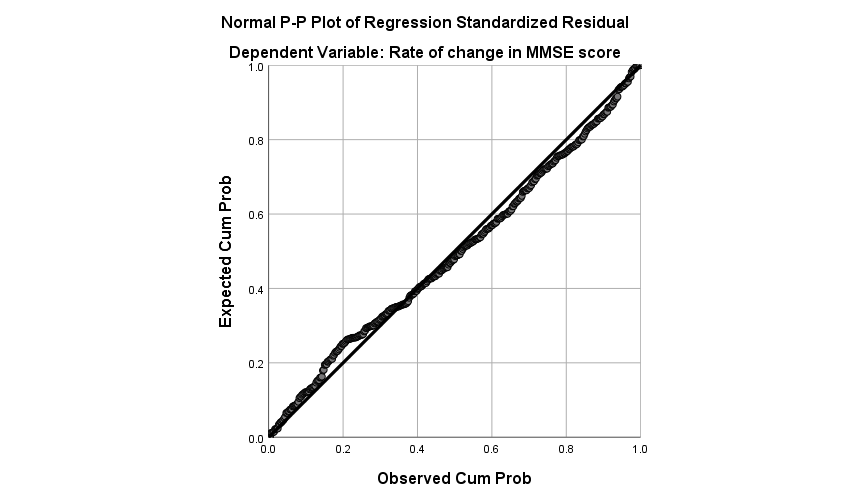
3.Male

4.Normal blood pressure
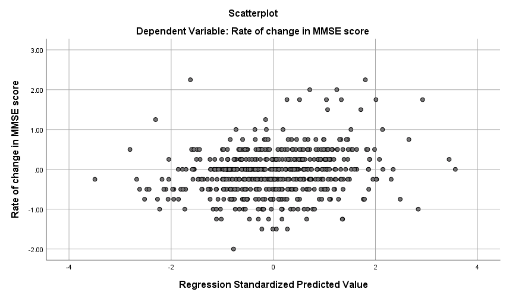

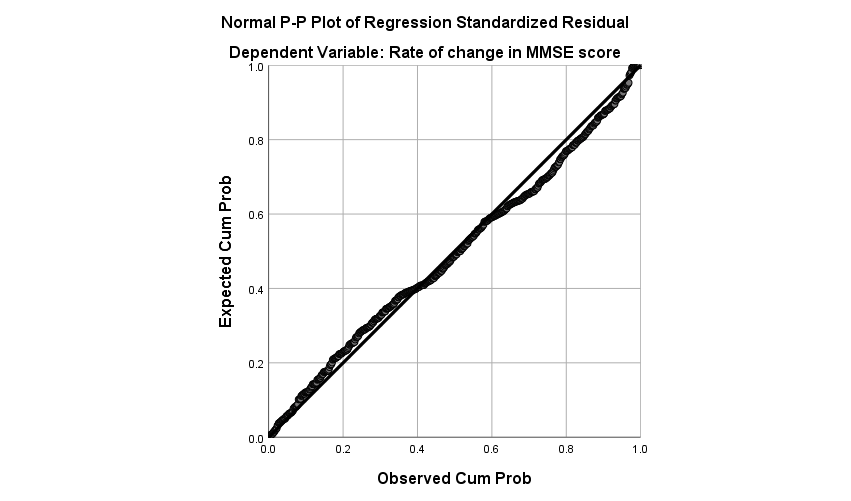


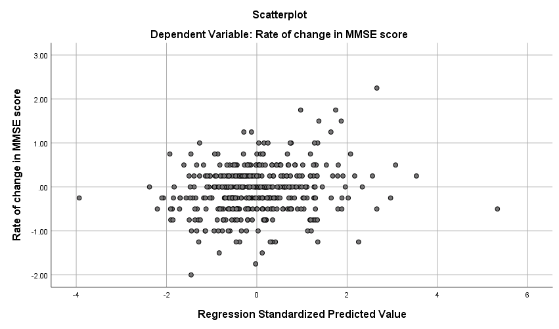
5.High blood pressure


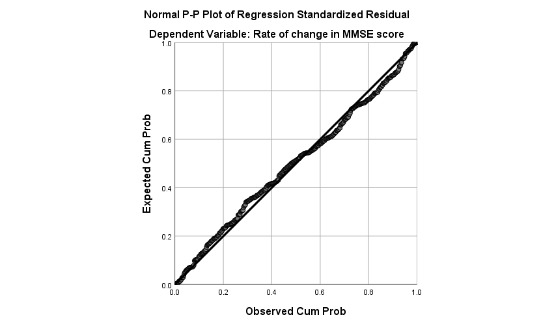


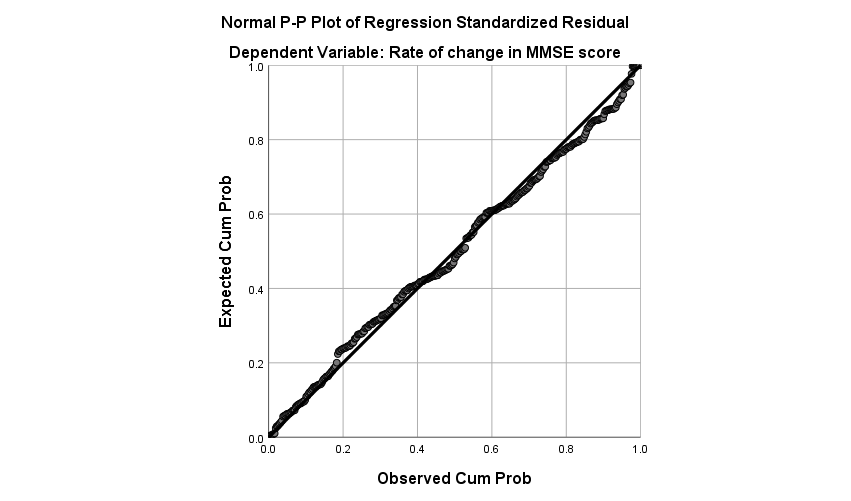

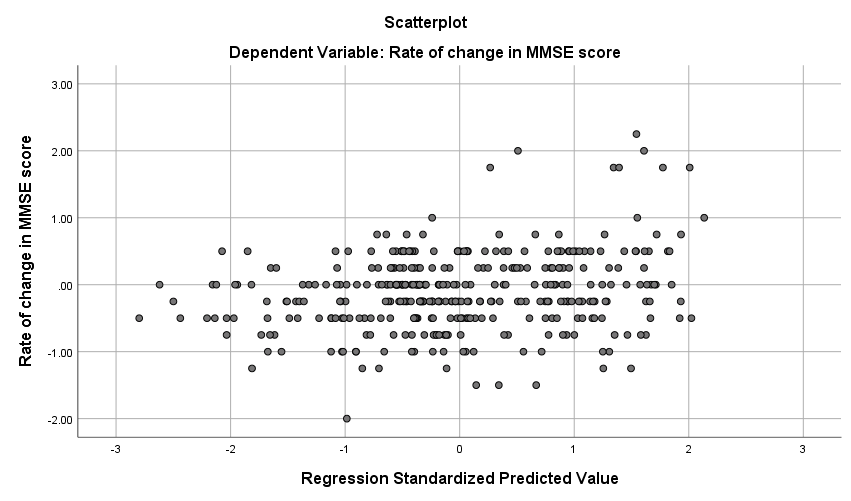
6.Normal BP-female


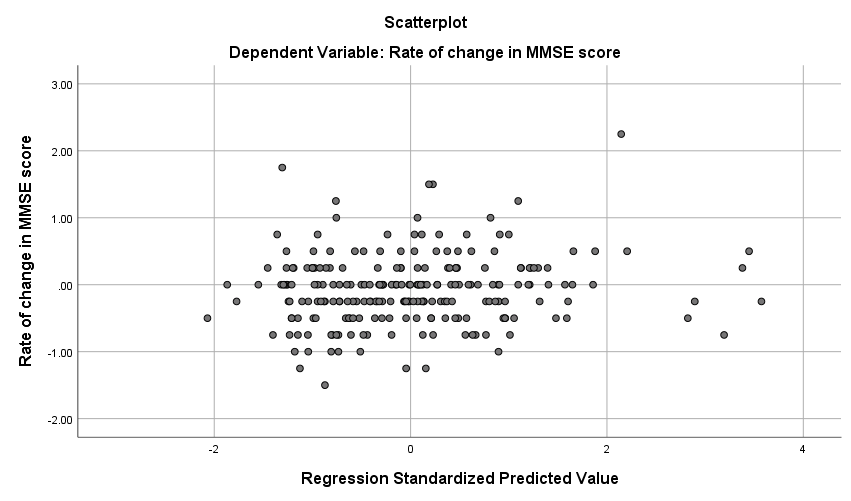
7.Normal BP-male


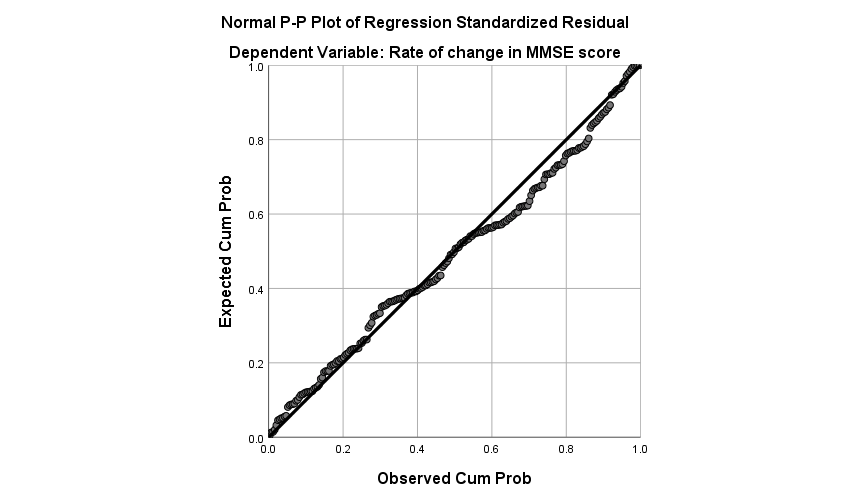


8.High BP-female


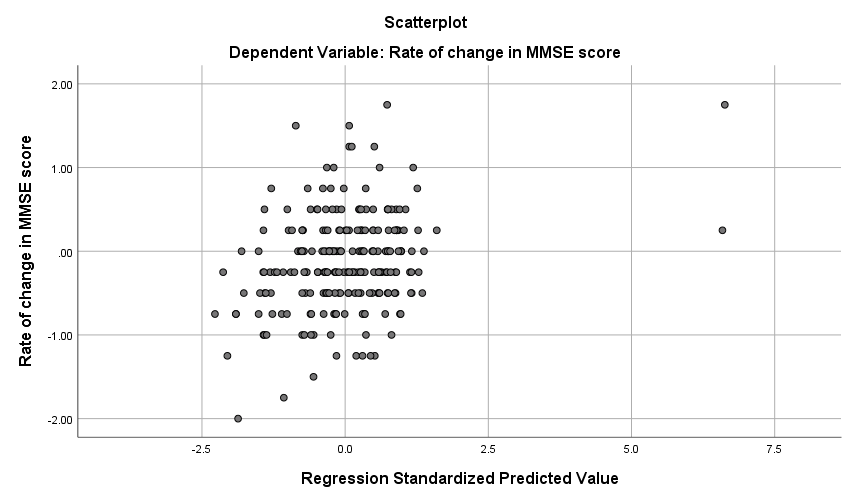

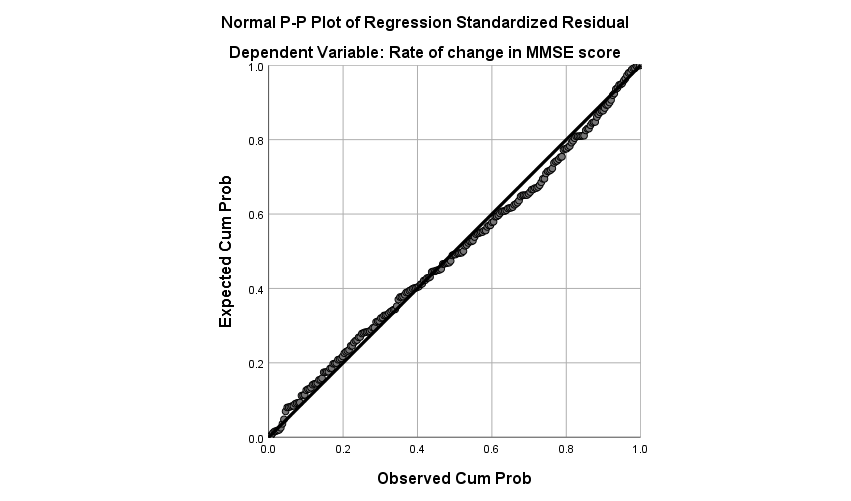


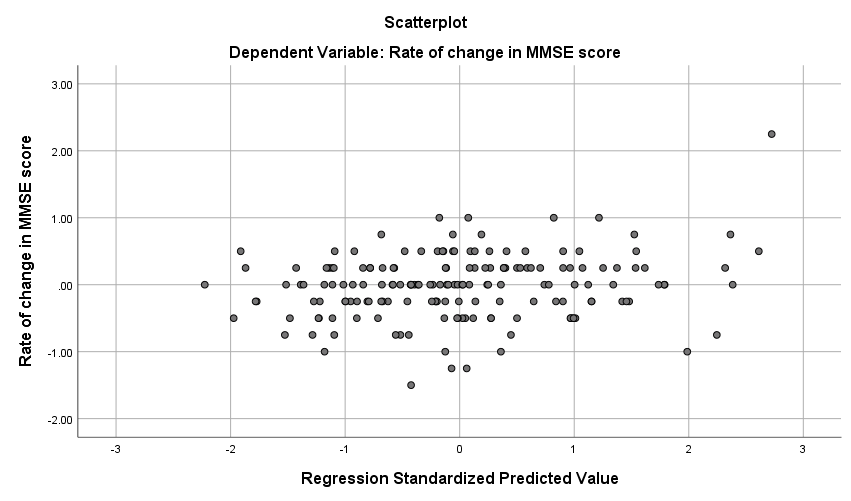

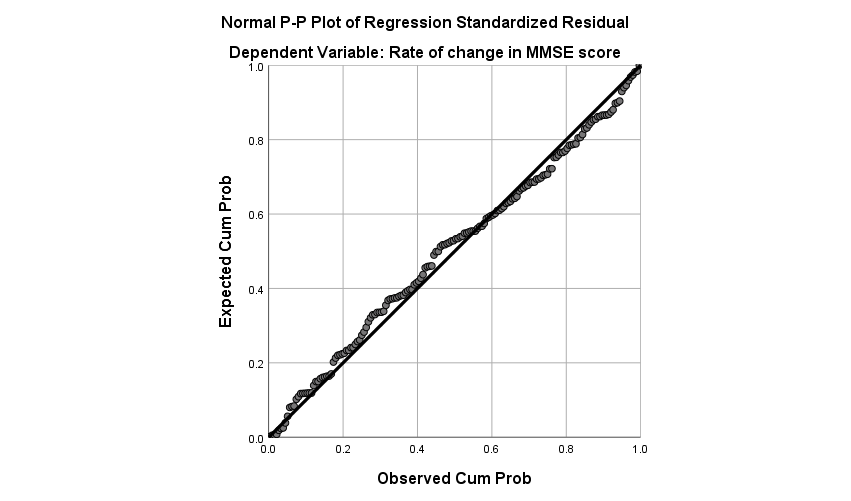
9.High BP-male
